# Severe orthostatic hypotension in otherwise uncomplicated *Plasmodium vivax* infection

**DOI:** 10.1186/s12936-020-03564-3

**Published:** 2021-01-07

**Authors:** Chaisith Sivakorn, Polrat Wilairatana, Srivicha Krudsood, Marcus J. Schultz, Tachpon Techarang, Khanittha Kheawsawaung, Arjen M. Dondorp

**Affiliations:** 1 Department of Clinical Tropical Medicine, Faculty of Tropical Medicine, Mahidol University, 420/6 Ratchawithi Road, Ratchathewi, 10400 Bangkok, Thailand; 2 Department of Tropical Hygiene, Faculty of Tropical Medicine, Mahidol University, Bangkok, Thailand; 3 Mahidol–Oxford Research Unit (MORU), Mahidol University, Bangkok, Thailand; 4 Department of Intensive Care, Academic Medical Center, and Laboratory of Experimental Intensive Care and Anesthesiology (L.E.I.C.A), University of Amsterdam, Amsterdam, The Netherlands; 5 Nuffield Department of Medicine, Oxford University, Oxford, UK; 6 School of Medicine, Walailak University, Nakhon Si Thammarat, Thailand; 7 Hospital for Tropical Diseases, Faculty of Tropical Medicine, Mahidol University, Bangkok, Thailand

**Keywords:** *Plasmodium vivax*, Orthostatic hypotension, Shock

## Abstract

Impaired autonomic control of postural homeostasis resulting in orthostatic hypotension has been described in falciparum malaria. However, severe orthostatic intolerance in *Plasmodium vivax* has been rarely reported. A case of non-immune previously healthy Thai woman presenting with *P. vivax* infection with well-documented orthostatic hypotension is described. In addition to oral chloroquine and intravenous artesunate, the patient was treated with fluid resuscitation and norepinephrine. During hospitalization, her haemodynamic profile revealed orthostatic hypotension persisting for another three days after microscopic and polymerase chain reaction confirmed parasite clearance. Potential causes are discussed.

## Background

Impaired autonomic control of postural homeostasis resulting in orthostatic hypotension has been described in falciparum malaria, but severe orthostatic intolerance in *Plasmodium vivax* has been rarely reported [1–3]. Orthostatic hypotension is defined as a decrease in systolic blood pressure of 20 mmHg or a decrease in diastolic blood pressure of 10 mmHg within 3 min after a change from a reclining to upright posture, together with associated symptoms [4]. It can be a debilitating symptom in conditions characterized by autonomous nervous system dysfunction [5]. Symptoms are caused by decreased cerebral perfusion, including blurred vision, fatigue, dizziness, and, in the most extreme cases, syncope [6]. On standing, the gravitational volume shift causes a redistribution of circulating blood, with pooling in the capacitance vessels below the diaphragm [7]. A normal compensatory haemodynamic response to changes in posture requires normal function of the cardiovascular, endocrine, and autonomic nervous systems [4]. Preservation of an adequate blood pressure is ensured by a prompt rise in cardiac output, mainly through an increase in heart rate, and an increase in vascular resistance favoring the cerebral blood circulation [8]. In dysautonomic states, this response to circulatory redistribution is impaired, which may lead to a compromised cerebral blood flow. Orthostatic hypotension in acute *Plasmodium falciparum* infection has been well described and is related to a combination of persisting relative bradycardia and insufficient peripheral vasoconstriction [9]. A case of severe orthostatic hypotension in a patient with a *P. vivax* infection, mimicking severe malaria is described. Haemodynamic profiles and possible pathophysiological features are discussed.

## Case presentation

Patient is a 32-year old previously healthy Thai female without a history of malaria and not taking any medication before the disease episode. Two weeks prior to admission she had travelled to a malaria endemic forested area in Kanchanaburi Province, Thailand for camping and hiking with friends and her 6-year-old son. Eight days prior to admission she developed a high-grade fever with headache and chills without localizing symptoms. She had a history of poor appetite, nausea and reduced oral intake without vomiting or diarrhoea. On admission, she had a fever of 39.8 °C, a pulse rate of 78 beats per minute and a blood pressure of 90/60 mmHg. Her weight was 49 kg which were 1 kg lower from her base line of 50 kg. Her consciousness was normal, conjunctivae were not pale and sclerae were not icteric. She had no cold or clammy skin and her capillary refill time was less than 2 s. The liver span was 10 cm in the mid-clavicular line and the spleen was normal in size on palpitation.

Initial complete blood count revealed anaemia with 31% haematocrit, as well as thrombocytopenia of 74,000/µL. Liver function tests revealed mild elevated aspartate aminotransferase and alanine aminotransferase of 60 U/L and 86 U/L, respectively. Blood sugar, creatinine, glucose-6-phosphate dehydrogenase (G6PD) enzyme activity and urinary analysis were normal. Microscopic examination of a peripheral blood film showed an asexual stage *P. vivax* parasitaemia of 69,800 parasites/µL and the diagnosis was confirmed by a positive polymerase chain reaction (PCR) for *P. vivax*. In patients breathing spontaneously, inferior vena cava (IVC) collapsibility index measured by transthoracic echocardiogram is a predictor of fluid responsiveness when the value was more than 42% [10]. In this patient, IVC collapsibility index was 46%, which is predictive of a positive fluid responsiveness. She was admitted to the Hospital for Tropical Diseases and given 600 mg chloroquine orally and started on 5% dextrose in 0.9% Sodium Chloride infusion at a rate of 80 mL/h. After 10 h and infusion of 800 mL fluids, the patient complained of postural faintness while getting out of bed. At that moment, she had an upright blood pressure of 77/46 mmHg and a pulse rate of 103 beats/min which increased to 90/50 mmHg in the supine position. Her urine output was 0.5 to 1 mL/kg per an hour. After lying down, she had good consciousness, warm extremities and capillary refill less than 2 s. Her IVC collapsibility index was 31%. Twelve-lead electrocardiogram showed sinus tachycardia with a normal QTc interval.

**Fig. 1:**
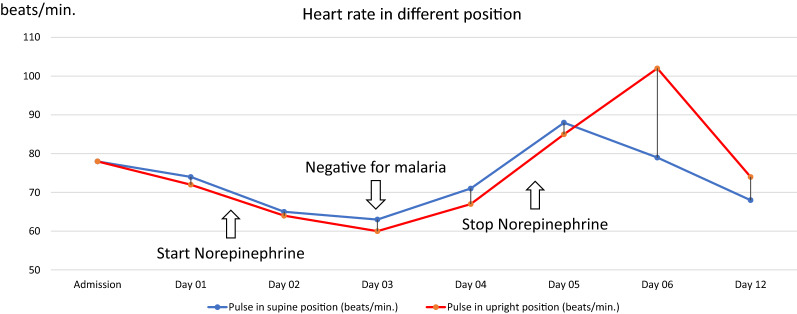
Daily heart rate in supine and standing for 3 min position

**Fig. 2:**
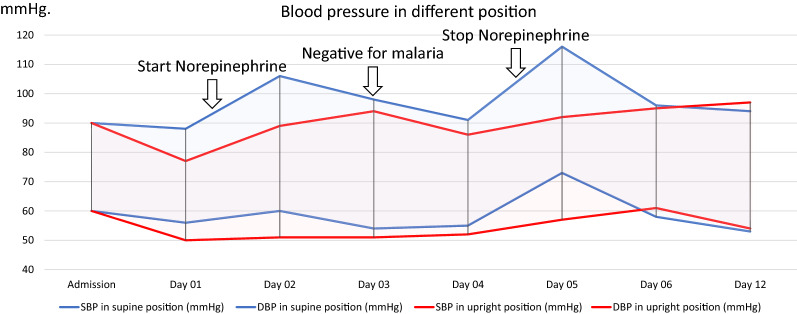
Daily systolic blood pressure and diastolic blood pressure in supine position and standing for 3 min position

**Fig. 3:**
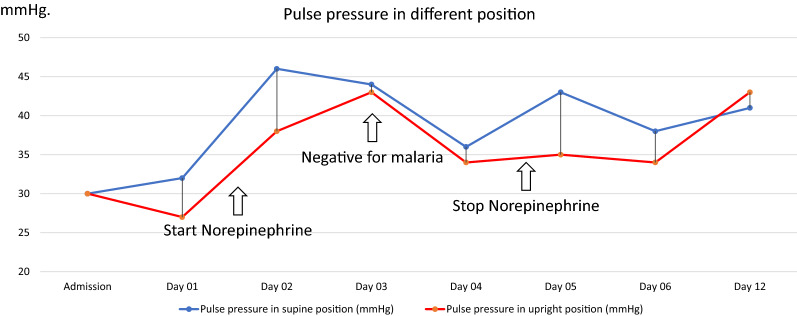
Daily pulse pressure in supine position and standing for 3 min position

**Fig. 4:**
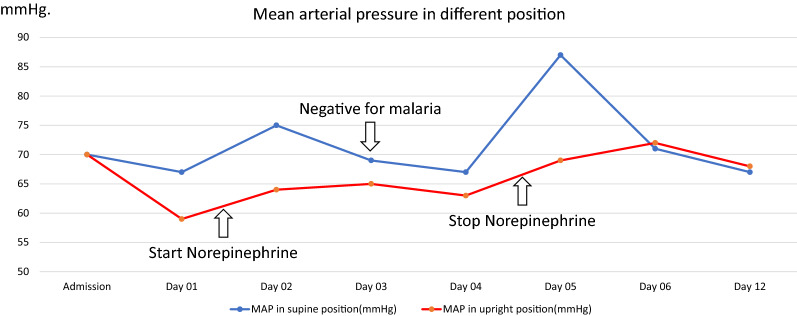
Daily mean arterial pressure in supine position and standing for 3 min position

**Fig. 5:**
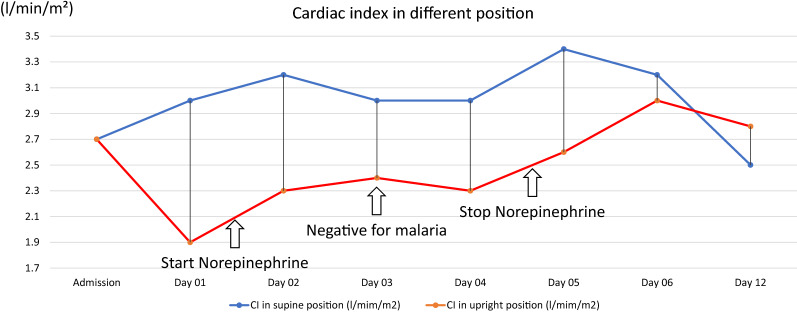
Daily cardiac index in supine position and standing for 3 min position

**Fig. 6:**
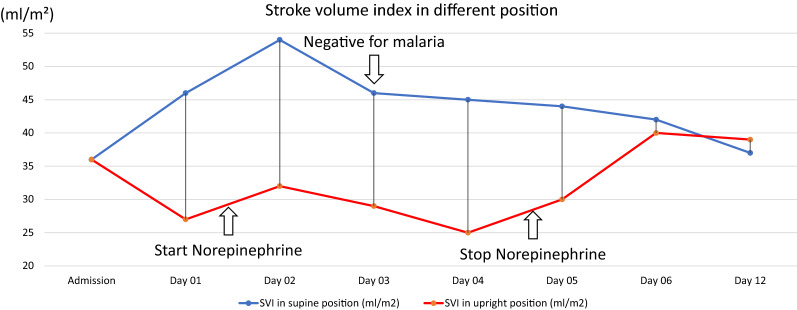
Daily stroke volume index in supine position and standing for 3 min position

She was initially diagnosed with severe *Plasmodium vivax* and given intravenous artesunate 2.4 mg/kg promptly followed by intravenous artesunate in the same dose after 12 h, which was repeated every 24 h for 5 days. Intravenous ceftriaxone was also started to cover potential concomitant bacterial septic shock awaiting blood culture results. Blood cultures obtained before start of antibiotics, however, remained without growth after which antibiotic therapy was discontinued on the 3rd day of admission. After transferring the patient to the intensive care unit, 400 mL normal saline was given over 1 h. After the fluid bolus, her blood pressure was 88/56 mmHg and her pulse rate was 74 beats/min in the supine position with falling to 77/50 mmHg and 72 beats/min while standing, and IVC collapsibility index of 16%, compatible with non-responsiveness to fluid resuscitation. Table 1 details her haemodynamic profiles during hospital admission. This prompted the start of intravenous norepinephrine at a dose of 0.13 µg/kg/min to maintain a blood pressure of 90/50 mmHg in an upright position. Her plasma lactate assessed at that moment was 1.8 mmol/L (normal value: < 2 mmol/L). Her morning serum cortisol on the next day was 32 µg/dL (normal level: > 6 µg/dL) making a diagnosis of primary or secondary adrenal insufficiency unlikely, and corticosteroids were not started. Haemodynamic monitoring by an ultrasound cardiac output monitor (USCOM) in the supine and upright position before receiving norepinephrine showed a marked drop in cardiac index from 3 L/min/m^2^ in the supine position to 1.9 L/min/m^2^ after standing for 3 min, despite adequate hydration. The upright positional drop in cardiac output in the upright position was explained by an absence of an increase in stroke volume, and an insufficient increase in heart rate (Table 1). This orthostatic hypotension persisted until 3 days after microscopically confirmed parasite clearance, necessitating continued vasopressor support with norepinephrine (Table 1) (Figs. 1, 2, 3, 4, 5 and 6). Orthostatic intolerance only resolved completely at the 12th day of follow up. After recovery, the patient received radical treatment with a 14-day course of (0.25 mg/kg daily) primaquine.

**Table 1:** Daily clinical data, laboratories and haemodynamic profiles

Day of admission	Admission	Day 01		Day 02		Day 03		Day 04		Day 05		Day 06		Day 12	
Position	Supine	Supine	Standing 3 min	Supine	Standing 3 min	Supine	Standing 3 min	Supine	Standing 3 min	Supine	Standing 3 min	Supine	Standing 3 min	Supine	Standing 3 min
Temperature (°C)	39.8	39.4		38		36.8		36.5		36.8		37		36.8	
Malaria															
Microscopic exam (parasites/µL)	430	155		62		Negative		Negative		Negative		Negative		Negative	
PCR	*Plasmodium vivax*	N/A		N/A		N/A		Negative for malaria		N/A		N/A		N/A	
Norepinephrine dose (µg/kg/min)	0	0.13		0.08		0.04		0.03		–		–		–	
Hemodynamic parameters															
IVC maximum (cm)	1.23	1.72	N/A	1.57	N/A	1.73	N/A	1.38	N/A	1.13	N/A	1.23	N/A	1.5	N/A
IVC minimum (cm)	0.66	1.18	N/A	1.23	N/A	1.38	N/A	1.03	N/A	0.84	N/A	1.08	N/A	0.98	N/A
IVC collapsibility (%)	46.34	31.39	N/A	21.7	N/A	20.23	N/A	25.36	N/A	25.66	N/A	12.2	N/A	34.67	N/A
Stroke volume index (mL/m²)	36	46	27	54	32	46	29	45	25	44	30	42	40	37	39
Cardiac index (L/min/m²)	2.7	3	1.9	3.2	2.3	3	2.4	3	2.3	3.4	2.6	3.2	3	2.5	2.8
Laboratories															
Hematocrit (%)	31.2			26.3		26.9		27.2		28.9		–		30	
Platelet (/µL)	74,000			68,000		117,000		158,000		213,000		–		240,000	
AST/ALT (U/L)	60/86			40/62		105/160		–		80/154		–		24/42	
Lactate (mmol/L)	1.8			1.3		0.99		–		–		–		–	
Intake/output	1,584/1,100			3248/1618		2761/3630		1593/2335		1387/1860		–		–	

*PCR* polymerase chain reaction, *IVC* inferior vena cava, *AST* aspartate aminotransferase, *ALT* alanine aminotransferase, *N/A* not available

IVC collapsibility = (maximum diameter − minimum diameter)/(maximum diameter) × 100

## Discussion

Hypotension meeting World Health Organization criteria for severe malaria has been reported in several case series of adult vivax malaria [11–14]. However, orthostatic hypotension which has been previously well documented during an acute *P. falciparum* infection [9] has been rarely described in *P. vivax* [1–3]. This case report describes a 5-day episode of orthostatic hypotension in a non-immune Thai woman with vivax malaria after start of treatment with chloroquine and after adequate fluid resuscitation. Her orthostatic intolerance was unlikely to be caused by an underlying disease or medication. Blood cultures have a low sensitivity for detecting bacteraemia, which might not exclude concurrent bacterial sepsis. Gram negative/Salmonella coinfection has been previously reported in vivax malaria [15]. However, the observed hypotension was not considered as a feature of septic shock from bacterial infection or severe malaria, since there were no signs of tissue hypoperfusion, with a normal plasma lactate concentration and normal capillary refill time, and no other indications of organ failure. She did receive low-dose vasopressor therapy, mainly to maintain an adequate blood pressure in an upright position. In falciparum malaria three potential mechanisms of orthostatic intolerance have been proposed. The first mechanism is autonomic dysfunction causing relative bradycardia and impaired capacity to increase vascular resistance in the upright position, which will cause a drop in mainly the diastolic blood pressure in the upright position, as also observed in the presented case.

In healthy young adults, the immediate response to a change in position from supine to upright is characterized by a prompt rise in heart rate of about 15 to 30%, and in total vascular resistance of 30 to 40% [8]. The main sensory receptors involved in the orthostatic neural reflex adjustment are the arterial mechanoreceptors (baroreceptors) located in the aortic arch and carotid sinuses and mechanoreceptors located in the heart and lungs (cardiopulmonary receptors). The cardiopulmonary receptors act in concert with the arterial baroreceptors to affect the necessary adjustment in sympathetic nerve action [16]. In patients with falciparum malaria, autonomic dysfunction causing an impaired neural reflex with insufficient compensatory tachycardia and arteriolar vasoconstriction is thought to be the primary cause of orthostatic hypotension in these patients [17], and the subsequent increased subdiaphragmatic pooling of venous blood will subsequently cause a reduction in venous return resulting in a further reduction in stroke volume and thus cardiac output, exaggerating the orthostatic fall in blood pressure.

A second mechanism is blood flow redistribution due to venous vasodilation caused by fever and other factors [18], with larger proportions of blood going to skin and muscle and decreased proportions to liver and kidney [19]. Combined with autonomic failure, this can further reduce venous return and thus cardiac output. Intravascular hypovolaemia can also contribute. Dehydration is common in patients with (falciparum) malaria, as illustrated by the frequently observed increase in the urea/creatinine ratio, increased plasma osmolarity and decreased fractional excretion of sodium, in the presence of an adequate antidiuretic hormone response [20]. Dehydration in patients with malaria is usually caused by transpiration, vomiting or diarrhea, and inadequate fluid intake because of the acute illness. In our case, however, the patient had been adequately rehydrated, as illustrated by the normal IVC collapsibility index after fluid administration.

Chloroquine has also been described to cause hypotension [21, 22] and negative chronotropic effect [23]. Chloroquine decreases vascular resistance [24] through veno-vasodilation via the release of endothelial nitric oxide in the venous circulation [25]. Through vasodilatation, chloroquine may thus reduce both cardiac preload and afterload causing hypotension. Chloroquine also impairs adaptation of the heart rate via reduction in the firing of the spontaneous action potential of the so-called ‘funny current, causing bradycardia [23]. The oral administration of chloroquine could also have triggered an initial vasovagal reaction, but this would not explain the persisting orthostatic symptoms in this patient. Since her orthostatic symptoms persisted after parasite clearance, it is likely that chloroquine therapy contributed to the orthostatic hypotension in our patient, since chloroquine has a long plasma half-life, with an initial t_1/2_ between 150 and 290 h [26]. In the acute phase of her illness, the described factors which have been identified contributing to orthostatic hypotension in falciparum malaria could have played a role, but this is difficult to substantiate.

In conclusion, this case report shows that orthostatic hypotension may occur in patients with uncomplicated *P. vivax* infection. This could result from chloroquine therapy, whereas *P. vivax* induced autonomic dysfunction may also contribute.

## Data Availability

The data that support the findings of this study are available from Hospital for Tropical Diseases, but restrictions apply to the availability of these data and so are not publicly available. However, data are available from the authors upon reasonable request and with the permission of the institution.

## Abbreviations

G6PD: Glucose-6-phosphate dehydrogenase
PCR: Polymerase chain reaction
IVC: Inferior vena cava

## References

[1] Povinelli L, Monson TA, Fox BC, Parise ME, Morrisey JM, Vaidya AB (2003). *Plasmodium vivax* malaria in spite of atovaquone/proguanil (malarone) prophylaxis. J Travel Med.

[2] Mohapatra M, Padhiary K, Mishra D, Sethy G (2002). Atypical manifestations of *Plasmodium vivax* malaria. Indian J Malariol.

[3] Upreti V, Gera V, Chamania L, Shetty R, Chopra M (2006). Malaria-the master masquerader. Med J Armed Forces India.

[4] Lanier JB, Mote MB, Clay EC (2011). Evaluation and management of orthostatic hypotension. Am Fam Physician.

[5] Nilsson D, Sutton R, Tas W, Burri P, Melander O, Fedorowski A (2015). Orthostatic changes in hemodynamics and cardiovascular biomarkers in dysautonomic patients. PLoS ONE.

[6] Moya A, Sutton R, Ammirati F, Blanc J-J, Brignole M, Dahm JB (2009). Guidelines for the diagnosis and management of syncope (version 2009): the Task Force for the Diagnosis and Management of Syncope of the European Society of Cardiology (ESC). Eur Heart J.

[7] Fedorowski A, Melander O (2013). Syndromes of orthostatic intolerance: a hidden danger. J Intern Med.

[8] Smith JJ, Porth CM, Erickson M (1994). Hemodynamic response to the upright posture. J Clin Pharmacol.

[9] Butler T, Weber DM (1973). On the nature of orthostatic hypotension in acute malaria. Am J Trop Med Hyg.

[10] Long E, Oakley E, Duke T, Babl FE (2017). Does respiratory variation in inferior vena cava diameter predict fluid responsiveness: a systematic review and meta-analysis. Shock.

[11] Kochar DK, Saxena V, Singh N, Kochar SK, Kumar SV, Das A (2005). *Plasmodium vivax* malaria. Emerg Infect Dis.

[12] Kochar DK, Das A, Kochar SK, Saxena V, Sirohi P, Garg S (2009). Severe *Plasmodium vivax* malaria: a report on serial cases from Bikaner in northwestern India. Am J Trop Med Hyg.

[13] Alexandre MA, Ferreira CO, Siqueira AM, Magalhães BL, Mourão MPG, Lacerda MV (2010). Severe *Plasmodium vivax* malaria, Brazilian Amazon. Emerg Infect Dis.

[14] Barber BE, William T, Grigg MJ, Menon J, Auburn S, Marfurt J (2013). A prospective comparative study of knowlesi, falciparum, and vivax malaria in Sabah, Malaysia: high proportion with severe disease from *Plasmodium knowlesi* and *Plasmodium vivax* but no mortality with early referral and artesunate therapy. Clin Infect Dis.

[15] Bhattacharya SK, Sur D, Dutta S, Kanungo S, Ochiai RL, Kim DR (2013). Vivax malaria and bacteraemia: a prospective study in Kolkata, India. Malar J.

[16] Rowell L, Rowell L (1993). Neural-humoral adjustments to orthostasis and long-term control. Human cardiovascular control.

[17] Smit AA, Halliwill JR, Low PA, Wieling W (1999). Pathophysiological basis of orthostatic hypotension in autonomic failure. J Physiol.

[18] Cooper KE (1994). Some responses of the cardiovascular system to heat and fever. Can J Cardiol.

[19] Rowell LB, Brengelmann GL, Blackmon JR, Murray JA (1970). Redistribution of blood flow during sustained high skin temperature in resting man. J Appl Physiol.

[20] Hanson J, Hossain A, Charunwatthana P, Hassan MU, Davis TM, Lam SW (2009). Hyponatremia in severe malaria: evidence for an appropriate anti-diuretic hormone response to hypovolemia. Am J Trop Med Hyg.

[21] Looareesuwan S, White N, Chanthavanich P, Edwards G, Nicholl D, Bunch C (1986). Cardiovascular toxicity and distribution kinetics of intravenous chloroquine. Br J Clin Pharmacol.

[22] Supanaranond W, Davis T, Pukrittayakamee S, Nagachinta B, White N (1993). Abnormal circulatory control in falciparum malaria: the effects of antimalarial drugs. Eur J Clin Pharmacol.

[23] Capel RA, Herring N, Kalla M, Yavari A, Mirams GR, Douglas G (2015). Hydroxychloroquine reduces heart rate by modulating the hyperpolarization-activated current if: novel electrophysiological insights and therapeutic potential. Heart Rhythm.

[24] Anigbogu C, Adigun S, Inyang I, Adegunloye B (1993). Chloroquine reduces blood pressure and forearm vascular resistance and increases forearm blood flow in healthy young adults. Clin Physiol.

[25] Abiose AK, Grossmann M, Tangphao O, Hoffman BB, Blaschke TF (1997). Chloroquine-induced venodilation in human hand veins. Clin Pharmacol Ther.

[26] Krishna S, White NJ (1996). Pharmacokinetics of quinine, chloroquine and amodiaquine. Clin Pharmacokinet.

